# Functionalized Lipid Nanoparticles for Targeted RNA Delivery in Immune and Inflammatory Diseases

**DOI:** 10.3390/biomedicines14050957

**Published:** 2026-04-22

**Authors:** Yeongji Jang, Hyun Kyu Song, Man Kyu Shim, Yoosoo Yang

**Affiliations:** 1 Medicinal Materials Research Center, Biomedical Research Division, Korea Institute of Science and Technology (KIST), Seoul 02792, Republic of Korea; yjjaaang@gmail.com (Y.J.); mks@kist.re.kr (M.K.S.); 2 Department of Life Sciences, Korea University, Seoul 02841, Republic of Korea; hksong@korea.ac.kr; 3 Department of Integrative Biotechnology, Sungkyunkwan University, Suwon 16419, Republic of Korea

**Keywords:** functionalized lipid nanoparticles, RNA delivery, targeted nanomedicine, immune cell targeting

## Abstract

Lipid nanoparticles (LNPs) have become an important platform for the delivery of RNA therapeutics, including messenger RNA (mRNA) and small interfering RNA (siRNA). However, most clinically approved LNP formulations exhibit strong liver tropism following systemic administration, which limits efficient delivery to extrahepatic tissues. This inherent biodistribution profile has therefore been recognized as a key challenge for expanding the therapeutic applications of RNA nanomedicine. Recent efforts have focused on engineering functionalized LNP systems to improve delivery specificity beyond the liver. Surface modification with targeting ligands—such as antibodies, peptides, and nucleic acid aptamers—can promote receptor-mediated uptake by specific immune cell populations, including macrophages, dendritic cells and T lymphocytes. In parallel, advances in lipid design have improved intracellular RNA delivery by facilitating endosomal escape. These developments have broadened the potential use of RNA nanomedicine for inflammatory disorders, including autoimmune diseases, neuroinflammation, and cardiovascular inflammation. Functionalized LNPs are also being investigated for in vivo engineering of immune cells. This review summarizes current strategies for designing functionalized LNP systems, highlights their emerging applications in immune and inflammatory diseases, and discusses key challenges for clinical translation.

## 1. Introduction

The rapid development and clinical deployment of mRNA-based COVID-19 vaccines have established lipid nanoparticles (LNPs) as a highly effective delivery platform for RNA therapeutics. RNA-based modalities—including messenger RNA (mRNA), small interfering RNA (siRNA), and antisense oligonucleotides (ASOs)—enable direct modulation of intracellular signaling pathways that are often inaccessible to conventional small-molecule drugs [1,2,3,4]. Unlike viral vectors or DNA-based approaches, RNA therapeutics do not integrate into the host genome and therefore provide transient and controllable modulation of gene expression. These properties make RNA delivery particularly attractive for diseases driven by dysregulated immune signaling. Despite these advantages, LNP systems still face inherent limitations, including a relatively short duration of gene expression and challenges in achieving precise cell-specific targeting compared with viral vectors such as adeno-associated viruses (AAVs). Nevertheless, LNPs offer key benefits, including improved safety profiles, lower risk of genomic integration, and greater flexibility in cargo design, making them an attractive alternative to viral and other delivery platforms.

Despite these advantages, most clinically validated LNP formulations exhibit strong accumulation in the liver following systemic administration [5,6]. This phenomenon is largely driven by interactions between nanoparticles and plasma proteins that form a protein corona on the nanoparticle surface. Among these proteins, apolipoprotein E (ApoE) plays a critical role by mediating the recognition of LNPs by low-density lipoprotein receptors expressed on hepatocytes [7,8,9,10]. Consequently, systemically administered LNPs are preferentially taken up by the liver. While this property has enabled several hepatic RNA therapies, it also represents a major limitation for achieving efficient RNA delivery to extrahepatic tissues.

Immune and inflammatory diseases frequently involve dysregulated activation of specific immune cell populations, including macrophages, dendritic cells, and lymphocyte subsets [11,12,13,14,15,16,17]. Effective RNA delivery in these settings requires overcoming multiple biological barriers. Nanoparticles must remain stable in circulation, cross vascular endothelium, and penetrate inflamed tissues before reaching target immune cells. In certain diseases, such as neuroinflammatory disorders, nanoparticles must also traverse highly restrictive biological barriers such as the blood–brain barrier (BBB) [18,19,20]. After cellular internalization, efficient escape from the endosomal compartment is required to prevent degradation of the RNA cargo in lysosomes [21]. To address these challenges, functionalized LNP platforms have been developed to improve targeting and intracellular delivery efficiency [22,23,24,25,26,27,28].

**Figure 1:**
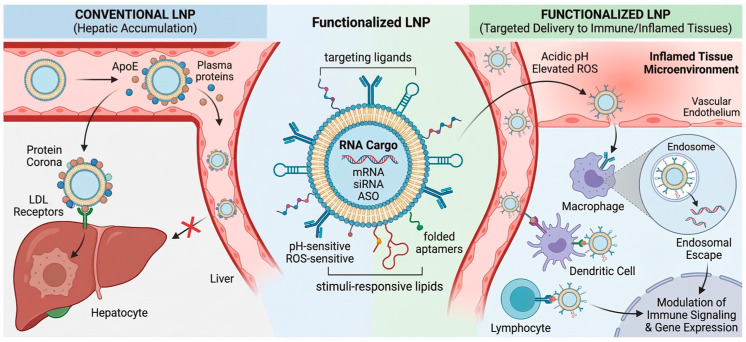
Design of Functionalized LNPs for Targeted RNA Delivery.

In this review, we summarize recent advances in the design of functionalized lipid nanoparticles for targeted RNA delivery (Figure 1). While existing literature often provides broad overviews of LNP delivery across diverse pathologies or focuses heavily on oncological applications, there remains a significant gap in synthesized knowledge regarding LNP optimization specifically for non-malignant immune and inflammatory contexts. These conditions present distinct physiological barriers, such as dynamic immune microenvironments and highly heterogeneous cell populations, which necessitate more precise spatio-temporal control over RNA delivery than conventional systemic approaches. This review specifically addresses these challenges by evaluating functionalization strategies tailored for immune-mediated disorders, thereby providing a specialized roadmap for the development of next-generation anti-inflammatory nanomedicines [29,30,31,32].

## 2. Design Strategies for Functionalized Lipid Nanoparticles

The therapeutic potential of LNPs for RNA delivery has expanded rapidly in recent years. However, most clinically validated LNP formulations exhibit strong liver tropism following systemic administration, which restricts their application to diseases involving other tissues [11,33,34]. To enable more precise delivery of RNA therapeutics, significant research efforts have focused on engineering functionalized LNP systems capable of targeting specific cell populations or pathological microenvironments [10,35].

**Figure 2:**
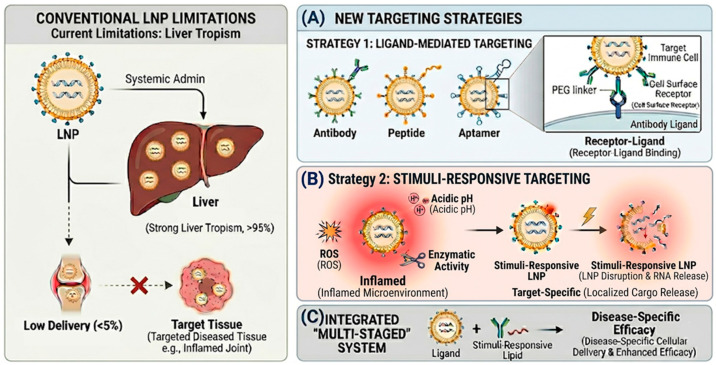
Comparative Overview of LNP Targeting Strategies. (**A**) Ligand-mediated targeting: Selective cellular uptake is achieved by decorating the LNP surface with various ligands (antibodies, peptides, or aptamers) that recognize specific receptors on target immune cells. (**B**) Stimuli-responsive delivery: LNPs are engineered to react to pathological microenvironments (e.g., acidic pH, high ROS levels, or specific enzyme overexpression). These stimuli trigger structural changes such as PEG-shedding or lipid destabilization, facilitating site-specific cargo release. (**C**) Combined ‘Multi-staged’ approach: Synergistic integration of ligands and stimuli-responsive elements. Surface ligands ensure initial cell-specific recruitment, while responsive components trigger sequential endosomal escape and intracellular RNA release.

Targeted RNA delivery using LNPs can generally be achieved through two complementary strategies (Figure 2). The first approach involves ligand-mediated targeting, in which nanoparticles are decorated with molecules that recognize specific cell-surface receptors. These ligand–receptor interactions promote receptor-mediated endocytosis and improve selective uptake by target cells. The second strategy relies on stimuli-responsive targeting, where nanoparticles are designed to respond to biochemical features of diseased tissues—such as acidic pH, oxidative stress, or enzyme activity—to trigger localized cargo release. Together, these methods offer a flexible way to deliver RNA directly to the specific immune cells and inflamed areas involved in a disease.

LNP formulations are typically composed of four main components: ionizable lipids, helper lipids, cholesterol, and PEG-lipids [36]. Ionizable lipids play a central role in RNA encapsulation and endosomal escape through pH-dependent charge transitions. Helper lipids, such as phospholipids, contribute to membrane structure and facilitate fusion with cellular membranes. Cholesterol improves nanoparticle stability and packing, while PEG-lipids regulate particle size, reduce aggregation, and extend circulation time. Crucially, the ratio and choice of these components determine the surface density and orientation of targeting ligands [37]. For instance, the concentration of PEG-lipids can modulate the accessibility of surface-conjugated ligands to their target receptors, while the overall lipid composition dictates the ‘biomolecular corona’ formation, which significantly impacts the in vivo targeting precision of functionalized LNPs.

### 2.1. Ligand-Mediated Targeting

Ligand-mediated targeting is one of the most widely explored strategies for improving the specificity of LNP-based RNA delivery [28]. In this approach, targeting ligands are attached to the nanoparticle surface to recognize receptors that are selectively expressed on particular cell types. The binding between the ligand and its receptor can facilitate receptor-mediated endocytosis, thereby enhancing nanoparticle uptake by target cells.

A variety of ligand classes have been explored for nanoparticle targeting, including antibodies, peptides, aptamers, and small molecules. The choice of ligand depends on multiple factors, such as binding affinity, molecular size, stability, and potential immunogenicity. In addition, the density and spatial orientation of ligands on the nanoparticle surface can strongly influence targeting efficiency.

#### 2.1.1. Monoclonal Antibodies and Fragments

Monoclonal antibodies are widely used as targeting ligands because of their high specificity and strong binding affinity [34,38,39]. Antibody–antigen interactions typically occur in the nanomolar to picomolar range, enabling precise recognition of target cells. In addition, the bivalent structure of antibodies can enhance binding through avidity effects, potentially increasing nanoparticle retention at the target site.

However, full-length antibodies (~150 kDa) substantially increase LNP size and may limit penetration into dense tissues [40,41]. Strong surface binding can also produce a “binding-site barrier,” in which nanoparticles accumulate near the outer layers of tissue rather than diffusing deeper into the target region. Moreover, the Fc domain of antibodies may interact with immune receptors and contribute to unintended immune activation or rapid clearance [42]. To address these limitations, smaller antibody-derived fragments such as Fab fragments (~50 kDa) and single-chain variable fragments (scFv, ~25 kDa) are increasingly used for nanoparticle targeting [43]. These engineered fragments retain antigen specificity while reducing steric hindrance and eliminating Fc-mediated immune interactions.

In immune and inflammatory diseases, ligand selection is typically based on cell-specific surface markers associated with disease pathology. Antibodies targeting CD206 have been used to direct LNPs to macrophages in inflamed tissues, while CD163-targeting antibodies enable delivery to anti-inflammatory macrophage subsets [44,45]. Antibodies against CD3 or CD8 have also been applied for targeting T cells in vivo [46]. These approaches reflect the need to match ligand selection with the dominant immune cell populations involved in each disease.

#### 2.1.2. Peptide and Small-Molecule Ligands

Peptide-based ligands provide a flexible alternative for targeted LNP delivery [20,22,47,48,49,50,51,52]. Short peptide sequences (typically 10–20 amino acids) can be displayed at relatively high density on LNP surfaces without significantly affecting particle size or stability. These peptides are commonly introduced through bioconjugation strategies, such as click chemistry-mediated coupling between functionalized lipids and peptide ligands, enabling stable presentation of the peptide on the LNP surface for receptor recognition [22]. Compared with protein-based ligands, peptides generally exhibit lower immunogenicity and can be produced using scalable chemical synthesis.

One limitation of peptide ligands is their relatively modest binding affinity for target receptors compared with larger biomolecules such as antibodies [53]. To compensate for this, LNPs frequently display multiple peptide ligands on their surface, allowing multivalent receptor interactions that increase overall binding strength through avidity effects. In this context, the spatial organization and surface density of peptide ligands are important determinants of targeting efficiency.

#### 2.1.3. Aptamer Ligands

Aptamers are short single-stranded DNA or RNA molecules capable of folding into three-dimensional structures that bind specific targets with high affinity [54,55,56]. Because they can achieve binding affinities comparable to those of antibodies, aptamers are sometimes described as “chemical antibodies”. Aptamers are typically identified through the SELEX (Systematic Evolution of Ligands by Exponential Enrichment) process [57,58]. Their relatively small size enables improved tissue penetration compared with large protein ligands. In addition, aptamers exhibit excellent batch reproducibility because they are produced through chemical synthesis rather than biological expression systems. Nevertheless, their susceptibility to nuclease degradation remains a challenge for in vivo applications. Chemical modifications such as 2′-fluoro or 2′-O-methyl substitutions are therefore frequently introduced to improve stability in biological fluids.

These ligand classes differ not only in size and binding affinity but also in their clinical feasibility [59]. Antibodies provide high specificity and established regulatory paths but may be limited by their large molecular size and potential immunogenicity. In contrast, peptides offer improved tissue penetration and lower production costs, although their lower individual affinity often necessitates high-density surface functionalization to achieve sufficient avidity. Aptamers present a unique balance between specificity and synthetic flexibility, but require advanced stabilization against degradation [60]. The selection of these ligands is not merely a matter of binding affinity; it involves a complex trade-off between molecular size, which dictates tissue penetration, and the ‘multivalent effect,’ where higher ligand density can compensate for lower individual affinity [61]. Thus, functionalization strategies must reconcile targeting precision with the physiological barriers of the target organ and the complexities of large-scale manufacturing.

### 2.2. Stimuli-Responsive Targeting

In addition to ligand-mediated recognition, targeted RNA delivery can also be achieved through stimuli-responsive LNP that exploit pathological features of diseased tissues. Many inflammatory and immune-related diseases are associated with distinct biochemical characteristics, including acidic microenvironments, elevated levels of ROS, and increased enzymatic activity [62,63,64]. LNPs engineered to respond to these conditions can enable spatially controlled activation or cargo release at the disease site. This strategy is particularly attractive for inflammatory diseases, where the microenvironment often differs substantially from that of healthy tissues. By incorporating stimuli-responsive materials into the LNP structure, LNP systems can achieve environment-triggered RNA release, thereby improving delivery specificity while reducing off-target effects.

#### 2.2.1. pH-Responsive Systems

Inflamed or diseased tissues frequently exhibit mildly acidic conditions compared with normal physiological environments. This feature has been widely exploited in the design of pH-responsive nanoparticle systems [65,66]. In these formulations, lipid components or linkers are engineered to undergo structural changes under acidic conditions, which can promote nanoparticle destabilization or trigger cargo release. For example, ionizable lipids used in many LNP formulations become protonated under acidic conditions within endosomes [67]. This charge conversion promotes interactions with negatively charged phospholipids in the endosomal membrane, facilitating membrane destabilization and enabling cytosolic release of RNA cargo.

#### 2.2.2. ROS-Responsive Systems

Elevated levels of reactive oxygen species are a hallmark of many inflammatory diseases [68]. Activated immune cells such as macrophages and neutrophils generate high concentrations of ROS as part of the inflammatory response. LNPs incorporating ROS-sensitive chemical groups can exploit this characteristic to enable selective activation within inflamed tissues [69]. ROS-responsive lipids or linkers may undergo oxidative cleavage in the presence of high ROS levels, leading to structural disruption of the LNP and release of the encapsulated RNA payload. Such systems offer a strategy for selectively enhancing RNA delivery in inflammatory microenvironments.

#### 2.2.3. Enzyme-Responsive Systems

Certain enzymes are overexpressed in diseased tissues and can serve as triggers for targeted drug release [70,71]. For example, matrix metalloproteinases (MMPs), esterases, and proteases are frequently upregulated in inflammatory lesions and tissue remodeling processes. In enzyme-responsive LNP systems, cleavable linkers are typically incorporated into lipid components or PEG–lipid conjugates within the LNP structure. These linkers can be designed as peptide or ester bonds that are specifically recognized by disease-associated enzymes. Upon enzymatic cleavage, structural changes such as PEG shedding or lipid destabilization can occur, which promotes cellular uptake or facilitates the release of encapsulated RNA cargo at the target site.

Furthermore, ligand-mediated and stimuli-responsive strategies are increasingly being integrated to create ‘multi-staged’ delivery systems [72]. In these hybrid approaches, surface ligands facilitate the initial recruitment and selective internalization of LNPs into target immune cells, while stimuli-responsive components—such as pH-sensitive or redox-cleavable lipids—trigger cargo release only upon reaching the acidic endosome or the reducing environment of the cytoplasm. This sequential activation not only enhances targeting specificity by providing dual layers of selectivity (extracellular recognition and intracellular triggering) but also significantly improves the endosomal escape efficiency, which remains a primary bottleneck in RNA delivery [73].

### 2.3. Bioconjugation Strategies for Ligand Attachment

The method used to attach targeting ligands to LNPs plays a critical role in determining the biological performance of functionalized systems [74,75,76]. Conjugation strategies must preserve ligand activity and maintain nanoparticle stability during systemic circulation. In addition, ligand orientation and surface density strongly influence receptor recognition and targeting efficiency.

An important consideration in ligand conjugation is site specificity [77]. Random chemical conjugation can generate heterogeneous ligand orientations and may partially block receptor-binding domains. Site-specific approaches, in contrast, allow ligands to be presented in a controlled orientation that preserves their biological activity.

#### 2.3.1. Thiol-Maleimide Conjugation

Thiol–maleimide chemistry is one of the most commonly used methods for ligand conjugation on lipid nanoparticles [78,79,80]. This reaction involves the nucleophilic addition of a thiol group—typically from a cysteine residue—to a maleimide-functionalized lipid, often incorporated into PEGylated lipid anchors. The reaction proceeds rapidly in aqueous conditions and generally provides high coupling efficiency. Despite these advantages, the stability of maleimide linkages in biological environments can be limited. In the presence of endogenous thiols such as glutathione or albumin, the thioether bond may undergo exchange reactions or retro-Michael reactions. Such instability can lead to gradual detachment of targeting ligands from the LNP surface during systemic circulation, potentially reducing targeting efficiency and increasing off-target accumulation.

#### 2.3.2. Copper-Free Click Chemistry

Bio-orthogonal click chemistry has emerged as a highly stable alternative for nanoparticle functionalization. In particular, strain-promoted azide–alkyne cycloaddition (SPAAC) between azide groups and cyclooctyne derivatives such as DBCO enables efficient ligand conjugation without the need for metal catalysts [81,82,83].

This reaction proceeds rapidly under physiological conditions and produces stable triazole linkages. Because azide and DBCO groups show minimal reactivity toward endogenous biomolecules, SPAAC provides excellent selectivity in complex biological systems [84]. As a result, this strategy has been widely used to attach peptides, antibodies, and nucleic acid ligands to nanoparticle surfaces while maintaining structural homogeneity and predictable pharmacokinetic behavior.

#### 2.3.3. Carbodiimide-Mediated Conjugation

Carbodiimide chemistry is also used for ligand conjugation on lipid nanoparticles. In this approach, 1-ethyl-3-(3-dimethylaminopropyl) carbodiimide (EDC) is typically combined with N-hydroxysuccinimide (NHS) to form amide bonds between carboxyl groups on lipid components and primary amines on targeting ligands [85,86]. Because many proteins and peptides contain accessible amine groups, EDC/NHS chemistry provides a straightforward route for ligand attachment. However, this strategy offers limited control over ligand orientation. Multiple reactive groups may be present on the ligand surface, and conjugation can occur at different positions. As a result, receptor-binding domains may be partially masked, leading to heterogeneous ligand presentation on the nanoparticle surface.

#### 2.3.4. Affinity-Based Ligand Attachment

In addition to covalent chemistry, affinity-based approaches have been explored for ligand presentation on LNP surfaces. These strategies rely on specific protein–protein interactions rather than direct chemical conjugation [87]. For example, Fc-binding domains such as Protein A or Protein G can capture antibodies through interactions with the Fc region. This interaction allows antibodies to be displayed on the nanoparticle surface while preserving the orientation of their antigen-binding domains. Alternative anchoring strategies have also been investigated to simplify antibody attachment to LNPs. In these systems, antibody–apolipoprotein fusion proteins associate with lipid membranes through the lipid-binding properties of apolipoproteins [23]. When mixed with LNPs, the fusion proteins form a stable protein corona on the particle surface and present the antibody outward. Because this approach avoids multistep chemical conjugation, it can simplify the functionalization process while maintaining antibody activity.

Taken together, these strategies establish a conceptual framework guided by four general design principles for next-generation functionalized LNP systems. First, ligand selection must be dictated by the specific expression kinetics and anatomical accessibility of receptors within inflamed tissues, moving beyond a narrow focus on binding affinity. Second, the combination of extracellular targeting and intracellular stimuli-responsive elements is essential for achieving the multi-layered selectivity required to bypass off-target effects in systemic circulation. Third, LNP lipid composition and surface functionalization must be optimized as a single unit, as the internal formulation directly dictates the orientation, density, and stability of surface ligands. Fourth, design choices—such as the use of affinity-based attachment or synthetic ligands—should prioritize scalability and stability from the outset to bridge the gap between benchtop innovation and clinical application. By adhering to these principles, researchers can move toward more predictable and cell-specific RNA delivery in complex immune landscapes.

## 3. Targeted RNA Delivery in Immune and Inflammatory Diseases

The design strategies discussed above provide the basis for directing LNPs toward specific immune cell populations [46,88]. On this foundation, functionalized LNP systems have been increasingly investigated as platforms for modulating immune responses in inflammatory and immune-mediated diseases (Table 1).

By delivering mRNA, siRNA, or other nucleic acid cargos to disease-relevant immune cells, LNPs can regulate cytokine production, reshape immune cell phenotypes, or transiently induce therapeutic receptor expression [89]. The following sections discuss representative cell-targeting strategies and their therapeutic applications in immune and inflammatory disorders.

The route of administration also influences the biodistribution and therapeutic performance of LNP systems [33]. Intravenous administration typically results in dominant liver accumulation due to interactions with plasma proteins such as ApoE, whereas intramuscular delivery promotes local expression and trafficking to draining lymph nodes, supporting immune activation [90]. Alternative routes have been explored to improve tissue-specific delivery. For example, intranasal administration has been investigated for neuroinflammatory diseases to partially bypass the blood–brain barrier, while local injection strategies can enhance delivery to inflamed tissues and reduce systemic exposure. These considerations highlight that the administration route is an important parameter in designing LNP-based therapies for different inflammatory conditions. For instance, while siRNA-mediated gene silencing is frequently employed via local delivery to dampen acute inflammation, mRNA-based protein replacement or vaccine strategies often utilize systemic or intramuscular routes to maximize therapeutic expression and immune engagement.

### 3.1. Cell-Specific Engineering for Immune Modulation

#### 3.1.1. Macrophages and Myeloid Cells

Macrophages are central regulators of inflammatory responses and are therefore important targets for RNA-based therapy [45,91]. Their marked functional plasticity allows them to adopt pro-inflammatory (M1-like) or anti-inflammatory (M2-like) states in response to local microenvironmental cues. In many chronic inflammatory disorders, persistent activation of M1-like macrophages drives cytokine production and contributes to tissue injury. Because macrophages are highly phagocytic, they are generally more amenable to LNP uptake than many other immune cell populations [92]. However, current strategies increasingly aim to target defined macrophage subsets rather than broadly modulating the entire myeloid compartment. This has led to growing interest in receptors that are preferentially expressed on macrophages within inflamed tissues.

One representative example is the mannose receptor, CD206, which has been widely used for macrophage-directed delivery [93,94]. Mannose-functionalized LNPs can bind CD206 and promote receptor-mediated uptake by macrophages. In several studies, mannosylated LNP formulations delivered anti-inflammatory mRNA or siRNA to macrophages and reduced the expression of cytokines such as TNF-α and IL-6 [95,96,97]. Another important target is CD163, a scavenger receptor associated with anti-inflammatory macrophage populations [44,98]. LNP systems functionalized with CD163-targeting ligands have been used to deliver RNA cargos that influence macrophage polarization. For example, delivery of IL-10 mRNA or related anti-inflammatory mediators can shift macrophages away from a pro-inflammatory phenotype toward a more regulatory state [99,100]. In addition to receptor-mediated targeting, microenvironment-responsive LNPs have been developed to enhance delivery selectivity within activated macrophages. In inflamed tissues, these cells are often exposed to elevated levels of ROS and intracellular glutathione [101]. LNP formulations incorporating ROS-sensitive linkers or disulfide-containing lipids can exploit these conditions to trigger intracellular cargo release after uptake. This design couples cell-associated targeting with stimulus-responsive release, thereby improving delivery specificity in inflammatory settings.

**Table 1:** Functionalized LNP systems for targeted RNA delivery with therapeutic potential in immune and inflammatory diseases.

Targeted Cell/Disease	RNA Cargo	Targeting Ligand/Design	Therapeutic Effect	Limitation	Ref.
Ly6C^+^ inflammatory leukocytes/IBD	*IRF8* siRNA	Anti-Ly6C antibody	Reduced IRF8 expression and decreased TNF-α, IL-6, IL-12/23, and IL-1β levels with improved colon pathology	Preclinical study with restricted cell-type specificity	[102]
Alveolar macrophages	*TAK1* siRNA	Anti-F4/80 antibody	Inhibition of NF-κB activation and reduced proinflammatory cytokine production with amelioration of lung injury	Limited cell specificity	[46]
Astrocytes	*TLR4* siRNA	Adenosine	Reduced TLR4 expression, decreased proinflammatory cytokines, increased anti-inflammatory cytokines, and attenuation of BBB disruption	Limited generalizability due to reliance on disrupted BBB conditions	[103]
Colonic macrophages	CRISPR/Cas9	Galactose	Reduced CD98 expression, decreased TNF-α and IL-6, increased IL-10, M2 macrophage polarization, and restoration of colonic barrier	Delivery efficiency variability	[104]
Lesional macrophages/atherosclerosis	*IL-10* mRNA	Mannose	Increased IL-10 expression, reduced plaque inflammation, decreased lipid deposition and necrotic area, and increased fibrous cap thickness	Preclinical validation only	[45]
Macrophages and chondrocytes	*miR-330-3p*	Folic acid, collagen II binding peptide	Reduced synovial inflammation, macrophage M1-to-M2 polarization, and enhanced cartilage repair	Preclinical validation and complex dual-target design	[105]
Microglia and astrocytes/Traumatic brain injury	p65 siRNA	Mannose, adenosine	Reduced neuroinflammation and BBB disruption	Preclinical focus and limited disease specificity	[106]

#### 3.1.2. T-Lymphocytes

T lymphocytes play a central role in adaptive immunity and are therefore attractive targets for RNA-based therapeutics [107,108]. However, efficient nucleic acid delivery to T cells remains challenging because these cells exhibit relatively low endocytic activity and are generally resistant to transfection compared with other immune populations.

These limitations require targeting strategies that actively promote cellular uptake [88,102]. LNP systems have been designed to engage T cell-specific surface markers to enhance receptor-mediated internalization. For example, antibody fragments such as scFv or Fab can be conjugated to the LNP surface to target markers including CD3, CD4, and CD8, enabling direct in vivo delivery of mRNA cargos to circulating T cells.

One prominent application is the in vivo generation of CAR-T cells [109,110]. Rather than engineering T cells ex vivo, LNPs can deliver mRNA encoding chimeric antigen receptors directly to circulating lymphocytes. In one study, CD5-targeted LNPs delivered CAR mRNA to T cells in vivo, generating engineered cells that recognized fibroblast activation protein (FAP) expressed on activated fibroblasts and reduced fibrosis in a mouse model of cardiac injury [111]. LNP platforms have also been investigated for transient expression of therapeutic receptors in T cells. Because mRNA expression is temporary, this strategy may reduce safety concerns associated with permanent genomic modification. Recent studies have further advanced this approach by using CD8-targeted LNPs to deliver anti-CD19 CAR mRNA, enabling in vivo T cell reprogramming, tumor control, and transient B cell depletion in nonhuman primates, suggesting a potential immune reset [112].

#### 3.1.3. Dendritic Cells

Dendritic cells (DCs) are specialized antigen-presenting cells that initiate and regulate adaptive immune responses [113,114]. Because DCs control the activation and differentiation of T cells, they represent important targets for RNA delivery strategies aimed at modulating immune activity in inflammatory and immune-mediated diseases. Targeted RNA delivery to DCs has therefore been explored to either stimulate protective immunity or induce immune tolerance. For example, DEC-205-targeted LNPs functionalized with a single-chain antibody have been used to selectively deliver siRNA to dendritic cells [115]. Gene silencing of costimulatory molecules such as CD40, CD80, and CD86 reduced dendritic cell-mediated T-cell activation. Another strategy focuses on targeting cross-presenting dendritic cell subsets through receptors such as CLEC9A. LNPs engineered with CLEC9A-binding ligands have been shown to enhance RNA delivery to cDC1 specialized in antigen cross-presentation, thereby improving T-cell priming and adaptive immune activation.

Beyond receptor targeting, LNP-mediated RNA delivery has also been explored to modulate DC function in inflammatory diseases [116,117]. For example, LNPs carrying autoantigen-encoding mRNA have been used to induce tolerogenic dendritic cells, which suppress autoreactive T-cell responses and promote the expansion of regulatory T cells (Tregs). Such strategies have demonstrated therapeutic potential in experimental models of autoimmune disease by restoring immune tolerance.

Despite these advances, delivery efficiency varies significantly across immune cell types [17,118,119]. For example, macrophages generally exhibit higher uptake due to their phagocytic activity, whereas T cells remain more difficult to transfect, requiring more sophisticated targeting strategies to overcome their minimal endocytic capacity [120]. These differences highlight the importance of tailoring LNP design according to the biological and anatomical characteristics of each immune cell population. From a translational perspective, successful DC targeting requires not only efficient cellular uptake but also precise intracellular trafficking to ensure that the delivered RNA reaches the appropriate compartment (e.g., cytoplasm for mRNA translation or endosomes for TLR modulation) without inducing unintended systemic immune activation [121]. Addressing these cell-specific bottlenecks is crucial for moving these platforms beyond preclinical efficacy toward consistent clinical performance.

### 3.2. Therapeutic Applications in Inflammatory Diseases

#### 3.2.1. Systemic and Localized Autoimmune Pathologies

Autoimmune diseases such as rheumatoid arthritis (RA) and systemic lupus erythematosus (SLE) are characterized by chronic immune activation that results in persistent inflammation and progressive tissue damage [88]. The pathological sites of autoimmune diseases, such as the synovial fluid in joints or the intestinal mucosa, exhibit distinct acidic microenvironments and enzyme overexpressions; these features provide the environmental rationale for stimuli-responsive LNPs to achieve site-specific cargo release. Effective RNA-based therapies require delivery systems capable of maintaining systemic stability while selectively targeting inflamed tissues and immune cell populations. Functionalized LNPs have emerged as promising platforms to achieve this goal by combining tissue-targeting ligands with environmentally responsive release mechanisms.

Rheumatoid arthritis provides a representative example of localized autoimmune inflammation. Arthritic synovial tissue is characterized by abnormal vascularization, hypoxia, and elevated oxidative stress, which together influence nanoparticle transport and cellular uptake [122]. These features create a microenvironment that can be exploited for targeted delivery. LNP systems have been designed to improve retention within inflamed joints and to enhance interactions with disease-associated immune cells. For example, hyaluronic acid-functionalized LNP formulations incorporated into MMP-responsive hydrogels enable enzyme-triggered release and CD44-mediated uptake by activated macrophages in arthritic synovium, resulting in reduced synovial inflammation and joint damage in preclinical models. Other LNP designs exploit the oxidative microenvironment of inflamed joints through ROS-responsive lipid components that promote stimulus-dependent cargo release within arthritic tissues [123].

Localized autoimmune inflammation is also central to inflammatory bowel disease (IBD), where dysregulated immune responses within the intestinal mucosa lead to chronic tissue injury. In this context, LNP-based RNA delivery strategies have focused on immune cells residing in the intestinal lamina propria and gut-associated lymphoid tissues. Mannose-functionalized LNPs, for example, have been used to target macrophages and dendritic cells expressing the mannose receptor (CD206), thereby enhancing uptake by intestinal immune cells [104]. Additional approaches have explored macrophage-directed delivery platforms based on ginsenoside-derived lipids that facilitate cellular uptake through glucose transporter-1-mediated recognition. When incorporated into colon-responsive microbead systems that protect siRNA during gastrointestinal transit, these LNP formulations enabled efficient delivery of TNF-α-targeting siRNA to intestinal macrophages and reduced inflammation in experimental colitis models [124].

In systemic autoimmune diseases such as SLE, pathogenic B cells and plasma cells play a central role in sustaining autoreactive immune responses [125]. Targeting these cell populations has therefore emerged as another strategy for LNP-mediated RNA therapy. For instance, LNP systems delivering mRNA encoding anti-CD19 antibodies have been developed to induce in vivo production of B-cell-depleting antibodies. In preclinical lupus models, this approach reduced the number of CD19^+^ B cells and plasma cells, leading to decreased tissue inflammation and improved disease pathology.

#### 3.2.2. Neuro-Renal Inflammation

Neuroinflammatory and renal diseases present additional delivery challenges because of strong anatomical barriers such as the BBB and the glomerular filtration barrier. To enable delivery across the BBB, functionalized nanoparticles have been designed to exploit receptor-mediated transcytosis pathways. For example, nanoparticles decorated with ligands targeting the transferrin receptor (TfR) have been used to improve transport across brain endothelial cells [126]. In experimental models, TfR-targeted LNPs have delivered RNA cargos to microglia, the resident macrophages of the central nervous system, and modulated inflammatory signaling associated with neurodegenerative diseases. In addition to microglia, astrocytes have also emerged as potential targets for RNA delivery in neuroinflammatory conditions [103]. Adenosine receptor-targeted LNP systems have been developed to facilitate astrocyte-specific siRNA delivery across the BBB. In a traumatic brain injury model, adenosine-functionalized LNPs enabled selective uptake by astrocytes and silencing of TLR4 signaling, resulting in reduced neuroinflammation and improved neurovascular protection.

Kidney-targeted RNA delivery presents a different set of challenges due to rapid filtration and the complex structure of renal tissues [127]. Recent studies have explored nanoparticles functionalized with ligands targeting podocyte receptors such as integrin α3β1 [128]. These systems have been used to deliver siRNA that suppresses oxidative stress pathways and fibrotic signaling in models of kidney injury.

#### 3.2.3. Cardiovascular and Metabolic Inflammatory Diseases

Chronic inflammation plays a central role in the progression of cardiovascular and metabolic diseases [129]. LNP-mediated RNA delivery has therefore been explored as a strategy to modulate inflammatory pathways and restore tissue homeostasis in these conditions by releasing RNA therapeutics locally in response to specific enzymes or oxidative stress.

In atherosclerosis, inflammatory macrophages accumulate within unstable plaques and contribute to disease progression and plaque instability [130,131]. Targeted LNP systems have been developed to deliver RNA therapeutics to plaque-associated macrophages in order to suppress pro-inflammatory signaling pathways. For example, LNP-mediated delivery of anti-inflammatory RNA cargos has been investigated to reduce macrophage-driven inflammation and improve plaque stability in experimental models of atherosclerosis.

Metabolic disorders such as obesity are also characterized by chronic inflammation within adipose tissue, which contributes to insulin resistance and metabolic dysfunction. Targeted RNA delivery strategies have therefore been investigated in tissues involved in systemic metabolic regulation [132]. For example, LNPs functionalized with skeletal muscle-specific receptor ligands targeting proteins such as LRP-3, MYH2, MYH4, MYF5, and MYOG have been used to deliver *miR-130a* selectively to myocytes. This targeted delivery reduced lipid deposition in skeletal muscle and improved metabolic parameters in diet-induced obese mice [133]. Liver-directed RNA delivery has also been examined using GalNAc-modified LNPs, which enable hepatocyte uptake through the asialoglycoprotein receptor (ASGPR). In one study, co-delivery of siRNA targeting dipeptidyl peptidase-4 (DPP-4) and mRNA encoding IL-27 modulated incretin signaling and thermogenic pathways in an obesity model, resulting in reduced body weight, adiposity, and circulating lipid levels [134].

## 4. Remaining Challenges and Future Perspectives

In recent years, many studies have explored functionalized LNPs to enable targeting beyond the liver, particularly for immune and inflammatory diseases. Despite these advances, several practical challenges remain. These include immune responses to nanoparticle components, differences between preclinical models and human physiology, and the difficulty of manufacturing complex targeted formulations at large scale.

### 4.1. Immune Responses to Nanoparticle Components

Immune recognition of nanoparticle components remains a concern, especially when repeated administration is required. PEG is commonly included in LNP formulations because it improves particle stability and prolongs circulation time [135,136,137]. However, antibodies against PEG have been reported in both untreated individuals and patients receiving PEGylated therapeutics.

The presence of anti-PEG antibodies can lead to accelerated blood clearance of PEGylated nanoparticles [138,139]. This effect reduces delivery efficiency and may increase the risk of infusion-related reactions. As a result, repeated dosing of PEG-containing LNPs may become less effective over time.

Several approaches have been explored to reduce this problem. Alternative polymers such as poly(sarcosine) and polysialic acid have shown reduced immune recognition while maintaining nanoparticle stability [139,140]. Another strategy uses PEG-lipids that gradually dissociate from the nanoparticle surface after injection [141,142]. This design maintains stability during circulation but reduces prolonged PEG exposure after delivery.

### 4.2. Clinical Translation Challenges

Many targeted LNP systems show promising results in animal studies but remain difficult to translate into clinical therapies [143,144]. One major limitation is the difference in nanoparticle behavior between animal models and humans. Targeting strategies that appear effective in mice often rely on receptor expression patterns that differ across species. For example, LNP uptake in the liver is strongly influenced by interactions with ApoE [9]. Although these mechanisms are well characterized in rodent models, variations in human plasma protein composition can lead to altered biodistribution profiles in clinical settings.

Repeated dosing also presents additional challenges [145,146]. Some RNA therapies require multiple administrations to maintain therapeutic effects, but repeated exposure to LNP formulations can induce immune responses against lipid components, including PEG. This may result in accelerated blood clearance and reduced delivery efficiency over time.

Manufacturing and formulation complexity further limit clinical translation. Functionalized LNP systems often involve multiple components or targeting ligands, which can increase batch-to-batch variability and complicate large-scale production. These factors contribute to the gap between preclinical efficacy and clinical performance. Addressing these translational hurdles requires a more sophisticated understanding of bio-nano interactions and the development of robust, scalable functionalization strategies.

Clinical experience with RNA therapeutics highlights these limitations. The approved siRNA drug patisiran uses a conventional LNP formulation that primarily targets the liver [147,148]. In contrast, achieving efficient and reproducible delivery to extrahepatic tissues remains a major challenge, and many targeted LNP systems have not yet demonstrated consistent performance in clinical settings.

### 4.3. Manufacturing and Product Consistency

Producing functionalized LNP systems at a large scale remains technically challenging [149,150]. In addition to RNA encapsulation, targeted nanoparticles require attachment of antibodies, peptides, or other ligands to the particle surface. These additional steps increase formulation complexity and may introduce variability in ligand density or orientation [26]. Small changes in ligand presentation can affect nanoparticle biodistribution and delivery efficiency. Maintaining consistent particle composition is therefore critical during manufacturing.

Microfluidic mixing methods have improved the reproducibility of LNP formulation by allowing controlled assembly of lipids and nucleic acids [27]. However, large-scale production still requires reliable analytical methods to measure particle size distribution, RNA encapsulation efficiency, and surface ligand content. Careful control of formulation and characterization will be important for regulatory approval of targeted LNP systems.

### 4.4. Emerging Approaches in RNA Nanomedicine

Recent efforts to improve RNA delivery have increasingly incorporated computational approaches for analyzing large formulation datasets [151,152]. Machine learning-based models can identify relationships between lipid structure, LNP composition, and delivery performance. Such approaches enable high-throughput systematic screening of lipid libraries and facilitate the identification of synergistic formulations with improved organ targeting or enhanced endosomal escape [153,154]. Furthermore, the integration of ‘Top-down’ protein corona engineering with ‘Bottom-up’ chemical functionalization represents a transformative shift toward predictable in vivo performance. Similar strategies have also been applied to optimize lipid compositions in selective organ targeting (SORT) LNP systems. In parallel, responsive lipid materials have been developed to react to local biological signals. Lipids sensitive to pH, redox conditions, or enzymatic activity can promote selective RNA release within inflamed or diseased tissues. Interactions between LNPs and plasma proteins also play an important role in determining biodistribution and cellular uptake. [155,156]. The protein corona formed in circulation can significantly influence LNP biodistribution and cellular uptake. A better understanding of these interactions may therefore guide the design of delivery systems with more predictable in vivo performance. Future advances in LNP technology will likely involve improved targeting strategies, next-generation lipid design, and integration with approaches such as gene editing and data-driven optimization [157]. These advances are expected to support the development of more precise and clinically translatable RNA therapeutics for immune and inflammatory diseases.

## 5. Conclusions

Lipid nanoparticles have become a key platform for RNA delivery and have enabled the development of several nucleic acid-based therapeutics. In recent years, efforts to functionalize LNPs with targeting ligands and responsive lipid components have expanded their potential beyond liver-directed delivery. In addition to mRNA-based approaches, LNP platforms have also been explored for the delivery of alternative nucleic acid cargos, such as circular RNA and DNA, which may enable more sustained or long-term gene expression [158,159].

In this review, we discussed recent strategies for engineering functionalized LNPs. These strategies aim to improve cellular targeting, enhance endosomal escape, and increase delivery efficiency in immune and inflammatory disease settings. Functionalized LNP systems are being explored in a range of disease contexts. Recent studies have also demonstrated the feasibility of in vivo immune cell engineering using LNP-mediated RNA delivery, such as transient CAR-T or CAR-NK cell generation.

Despite these advances, several challenges remain. Immune responses to nanoparticle components, differences between animal models and human physiology, and manufacturing complexity continue to limit clinical translation. Continued progress in nanoparticle design, improved understanding of nanoparticle–protein interactions, and advances in formulation technologies may help address these limitations. These efforts are expected to support the development of more effective RNA delivery systems for immune and inflammatory diseases.

## Data Availability

No new data were created or analyzed in this study. Data sharing is not applicable to this article.

## Abbreviations

The following abbreviations are used in this manuscript:

ASGPR	Asialoglycoprotein receptor
ASO	Antisense oligonucleotide
ApoE	Apolipoprotein E
BBB	Blood–brain barrier
CAR	Chimeric antigen receptor
CRISPR	Clustered regularly interspaced short palindromic repeats
DC	Dendritic cell
DEC205	Dendritic and epithelial cell 205
Fab	Fragment antigen-binding
Fc	Fragment crystallizable
FAP	Fibroblast activation protein
LNP	Lipid nanoparticle
MMP	Matrix metalloproteinase
mRNA	Messenger RNA
PEG	Polyethylene glycol
ROS	Reactive oxygen species
scFv	Single-chain variable fragment
SELEX	Systematic evolution of ligands by exponential enrichment
siRNA	Small interfering RNA
SORT	Selective organ targeting
TNF-α	Tumor necrosis factor alpha
Treg	Regulatory T cell
